# Adipocyte-specific blockade of gamma-secretase, but not inhibition of Notch activity, reduces adipose insulin sensitivity

**DOI:** 10.1016/j.molmet.2015.11.006

**Published:** 2015-12-02

**Authors:** David P. Sparling, Junjie Yu, KyeongJin Kim, Changyu Zhu, Sebastian Brachs, Andreas L. Birkenfeld, Utpal B. Pajvani

**Affiliations:** 1 Departments of Pediatrics, Columbia University, New York, NY 10032, USA; 2 Department of Medicine, Columbia University, New York, NY 10032, USA; 3 Department of Endocrinology, Diabetes and Nutrition, Center for Cardiovascular Research, Charité – University School of Medicine, Berlin, Germany; 4 Section of Metabolic Vascular Medicine, Medical Clinic III and Paul Langerhans Institute Dresden (PLID), a member of the German Center for Diabetes Research (DZD), Technische Universität Dresden, Germany; 5 Section of Diabetes and Nutritional Sciences, Rayne Institute, Denmark Hill Campus, King's College London, UK

**Keywords:** Notch, γ-secretase complex, Insulin resistance, 2DOG, 2-Deoxy-glucose, DBZ, Dibenzazepine, GSI, γ-secretase inhibitor, NICD, Notch intracellular domain, Rbp-Jκ, Recombination signal binding protein-Jκ, T2D, Type 2 diabetes

## Abstract

**Objective:**

As the obesity pandemic continues to expand, novel molecular targets to reduce obesity-related insulin resistance and Type 2 Diabetes (T2D) continue to be needed. We have recently shown that obesity is associated with reactivated liver Notch signaling, which, in turn, increases hepatic insulin resistance, opening up therapeutic avenues for Notch inhibitors to be repurposed for T2D. Herein, we tested the systemic effects of γ-secretase inhibitors (GSIs), which prevent endogenous Notch activation, and confirmed these effects through creation and characterization of two different adipocyte-specific Notch loss-of-function mouse models through genetic ablation of the Notch transcriptional effector Rbp-Jk (*A-Rbpj*) and the obligate γ-secretase component Nicastrin (*A-Nicastrin*).

**Methods:**

Glucose homeostasis and both local adipose and systemic insulin sensitivity were examined in GSI-treated, *A-Rbpj* and *A-Nicastrin* mice, as well as vehicle-treated or control littermates, with complementary *in vitro* studies in primary hepatocytes and 3T3-L1 adipocytes.

**Results:**

GSI-treatment increases hepatic insulin sensitivity in obese mice but leads to reciprocal lowering of adipose glucose disposal. While *A-Rbpj* mice show normal body weight, adipose development and mass and unchanged adipose insulin sensitivity as control littermates, *A-Nicastrin* mice are relatively insulin-resistant, mirroring the GSI effect on adipose insulin action.

**Conclusions:**

Notch signaling is dispensable for normal adipocyte function, but adipocyte-specific γ-secretase blockade reduces adipose insulin sensitivity, suggesting that specific Notch inhibitors would be preferable to GSIs for application in T2D.

## Highlights

• γ-secretase inhibitors (GSIs) are non-specific inhibitors of Notch signaling.
• GSI-treatment of obese mice increases hepatic, but lowers adipose insulin sensitivity.
• Adipocyte-specific Notch inhibition does not affect adipose mass or glucose homeostasis.
• Adipocyte-specific γ-secretase blockade reduces adipose insulin sensitivity.
• Specific Notch inhibitors may be preferable to GSIs for treatment of Type 2 Diabetes.

## 1 Introduction

Continued Westernization of diet and lifestyle in the setting of conducive genetics predispose to obesity, defined as excessive adipose mass [1]. Increased adiposity can then lead to insulin resistance, which predicts Type 2 Diabetes (T2D) [2]. Better understanding of the hormonal and mechanical signals underlying adipocyte-systemic crosstalk to induce insulin resistance is necessary to develop novel therapeutic targets to interrupt this burgeoning crisis.

The Notch cascade is a paracrine signaling pathway that has a well-established role in regulating normal differentiation by a complex process known as lateral inhibition [3]. Notch signaling is regulated post-translationally by ligand availability and multiple processing steps [4]. Notch receptors (Notch1-4) are activated by a transmembrane ligand of either the Jagged (Jagged1/2) or Delta-like (Dll-1/3/4) family on a neighboring cell, leading to a sequential cleavage by ADAM/TACE and the γ-secretase complex, releasing the soluble Notch intracellular domain (NICD). NICD translocates to the nucleus and activates Rbp-Jκ-dependent transcription of Notch targets, classically the *Hes* (Hairy and enhancer of split) and *Hey* (Hairy/enhancer-of-split related with YRPW motif) family of basic helix-loop-helix transcription factors, which regulate cell proliferation and embryogenesis and are indispensable for normal development [5]. More recently, Notch gain-of-function mutations have been associated with T-cell leukemia [6] and multiple solid tumors [7], leading to widespread development of Notch inhibitors as chemotherapeutic agents [8]. Of these, the most advanced are inhibitors of the γ-secretase (GSIs), a multi-protein complex consisting of catalytic (Presenilin 1 or 2), regulatory (PEN2 and Aph1a or 1b) and targeting (Nicastrin) subunits [9]. Although GSIs target numerous other Type-I transmembrane targets [10], including amyloid precursor protein (APP) [11], knockout of multiple γ-secretase subunits phenocopy the embryonic lethality of Rbp-Jκ deletion [5], [12], [13], underscoring the necessity of γ-secretase function for Notch activity.

We have recently shown that Notch plays a post-development role to regulate liver glucose and lipid metabolism [14], [15]. Liver-specific Rbp-Jκ deletion results in increased hepatic insulin sensitivity and improved glucose tolerance; consistently, GSI-treated obese mice show marked improvements in glucose tolerance [14]. These data have since been confirmed using other GSIs and more specific Notch antagonists [15], [16], [17], leading to the hypothesis that Notch signaling may be “re-activated”, and thus potentially targetable, in other tissues in the obese state. To address this question, we studied potential extra-hepatic effects of GSIs and found that while GSIs increase hepatic insulin sensitivity, they simultaneously reduce glucose uptake in white adipose tissue. To determine whether GSI-induced adipose insulin resistance was Notch-dependent, we created adipocyte-specific Rbp-Jk (henceforth, *A-Rbpj* mice) and γ-secretase (henceforth, *A-Nicastrin* mice) knockout mice, using the well-characterized Adiponectin-Cre transgenic mouse [18]. Although *A-Rbpj* and *A-Nicastrin* mice both develop normally, with unchanged body weight/adiposity as compared to Cre-littermates, *A-Rbpj* mice showed normal glucose homeostasis whereas *A-Nicastrin* mice showed a comparable reduction in adipocyte insulin sensitivity as GSI-treated mice. These data suggest that Notch activity is not required for normal adipocyte function but that γ-secretase activity regulates adipose insulin sensitivity, likely through a Notch-independent mechanism.

## 2 Materials and methods

### 2.1 Experimental animals

Male 8 week old *C57/BL6* mice were purchased from Jackson Laboratories. We intercrossed Adiponectin-cre [18] with *Nicastrin*^flox/flox^ [19] and *Rbpj*^*flox/flox*^ [15] mice to generate Adiponectin(cre):*Nicastrin*^flox/flox^ (*A-Nicastrin)* and Adiponectin(cre)*:Rbpj*^*flox/flox*^ (*A-Rbpj*) mice. All studies were performed using male mice. Mice were weaned to either standard chow (Purina Mills 5053) or high-fat diet (HFD) (18.4% calories/carbohydrates, 21.3% calories/protein and 60.3% calories/fat derived from lard; Harlan Laboratories, TD.06414). All animal procedures were approved by the Columbia University Institutional Animal Care and Utilization Committee.

### 2.2 Assays

Measurement of blood glucose (One Touch), plasma insulin (Millipore), and lipids were performed as previously described [20]. Intraperitoneal glucose tolerance tests (IP-GTT) were performed after a 16 h fast with 2 g/kg glucose. Body composition was measured by NMR (Bruker Optics).

### 2.3 Gamma-secretase inhibitor (GSI)

GSI was used as previously described [14]. In short, (S)-2-[2-(3,5-Difluoro-phenyl)-acetylamino]-N-((S)-5-methyl-6-oxo-6,7-dihydro-5H-dibenzo[b,d]azepin-7-yl)-propionamide, also known by the trade name dibenzazepine (DBZ), was suspended in vehicle [0.5% Methocel E4M (wt/vol, Colorcon) and 0.1% (vol/vol) Tween-80 (Sigma)] [21]. Immediately prior to injection, DBZ was sonicated for 2 min to suspension.

### 2.4 Glucose turnover studies

To measure glucose turnover and uptake in the fasting state, we omitted the insulin infusion during our standard glucose-insulin clamp protocol, as described previously [22]. In brief, awake mice with an indwelling catheter implanted in the right jugular vein one week before the experiment, were fasted overnight, and 3-[^3^H]glucose (Hartmann Analytical, Germany) was then infused at 0.05 ìCi/min for 120 min to determine basal glucose turnover. 10 ìCi of 2-deoxy-d-[1-^14^C]glucose (2DOG, Hartmann Analytical, Germany) was infused within 3 min to measure organ specific glucose uptake. Blood samples were drawn by tail vein at baseline and at 120 min after the initiation of the 2DOG infusion. At study completion, mice were anesthetized and tissues were harvested, snap frozen in liquid N_2_ within 3 min of collection using liquid N_2_-cooled tongs, and stored at −80 °C for subsequent analysis. Intracellular (6-phosphorylated) 2DOG uptake of epididymal white adipose tissue under basal conditions was measured as described [22].

### 2.5 Quantitative reverse-transcription PCR

RNA was isolated from adipose and liver with RNeasy Lipid and RNeasy mini-kits (Qiagen), respectively. cDNA was synthesized with qScript cDNA SuperMix (Quanta Biosciences), and quantitative PCR performed with a CFX96 Real-Time PCR detection system (Bio-Rad) and GoTaq SYBR Green qPCR kit (Promega) using the ΔΔC(t) method, with TATA-binding protein (TBP) and/or 18S as controls to determine relative gene expression.

### 2.6 Western blotting

3T3-L1 cells (ATCC) were differentiated per standard protocol. Day 8–10 adipocytes were incubated with 200 nM Compound E overnight, serum starved for 4 h, then treated with 100 nM bovine insulin (Sigma) for 15 min prior to lysis. Both 3T3-L1 lysates and whole adipose extracts were lysed in Adipose Lysis Buffer (20 mM Tris, pH 7.4 150 mM NaCl, 10% glycerol, 2% Nonidet P-40, 1 mM EDTA, pH 8.0, 0.1% SDS, 0.5% sodium deoxycholate, 20 mM NaF, 30 mM NaPPi, 1 mM NaVO4), supplemented with Complete Protease Inhibitor Cocktail Tablet, EDTA-free (Roche). Immunoblots were probed with antibodies against Nicastrin (#5665), Psen2 (#2192), phospho-Akt Thr308 (#9275), total Akt (#9272), phospho-GSK-3β Ser9 (#9322), total GSK-3β (#9315), tubulin (#2148), and actin (#8456) from Cell Signaling.

### 2.7 Statistical analysis

All results are reported as ± SEM unless otherwise indicated. Gene expression levels were compared using Students t-test. IP-GTT area under the curve was calculated using the trapezoidal rule. P values of <0.05 were considered significant.

## 3 Results

### 3.1 GSIs increase hepatic insulin sensitivity

**Figure 1:**
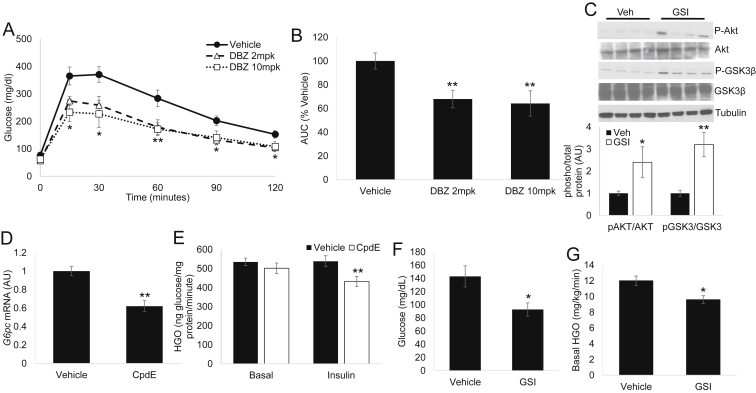
**γ-secretase inhibitors (GSIs) increase hepatic insulin sensitivity (A)** Glucose tolerance testing (GTT) of male, HFD-fed C57/BL6 mice, after 5 daily injections of vehicle or Dibenzazepine (DBZ) at either 2 mcg per kg body weight (2mpk) or 10 mcg per kg body weight (10mpk) doses, and **(B)** area under the curve (AUC) during GTT, normalized to vehicle-dosed mice (n = 6 mice/group). **(C)** Western blots (top) and quantification of pAKT and pGSK3β protein levels (normalized to total Akt or GSK3β signal, bottom) from livers of Vehicle- or GSI (DBZ 2mpk)-treated, HFD-fed C57/BL6 mice sacrificed after a 16 h fast followed by 4 h refeeding. **(D)***Glucose-6-phosphatase* (*G6pc*) expression, and **(E)** glucose output from primary hepatocytes treated with vehicle or Compound E (CpdE), with or without 75pM insulin treatment. Data shown are representative of 3 independent experiments. **(F)** Plasma glucose and **(G)** basal hepatic glucose output (HGO) in vehicle- or GSI-treated, HFD-fed C57/BL6 mice (n = 9–11 mice/group). *P < 0.05 and **P < 0.01 vs. Vehicle.

We have previously shown that dibenzazepine (DBZ), a well-characterized, bioavailable Notch inhibitor of the GSI class [21], [23], improves glucose tolerance in diet-induced or leptin-deficient (*ob/ob*) obese mice [14] but results in dose-limiting intestinal metaplasia [23]. To determine if a therapeutic window exists for safe application of this class of drugs for metabolic disease, we performed a dose-finding study. Interestingly, “low-dose” (2 mcg per kg body weight) DBZ treatment showed comparable potency to improve glucose tolerance as the previously used “high-dose” (10 mcg per kg body weight) (Figure 1A) without apparent intestinal toxicity (Supplemental Figure 1A, B). Neither dose altered food intake, adipose, or body weight (not shown, Supplemental Figure 1C, D). These data suggest differential susceptibility across tissues to Notch inhibition, and we used low-dose DBZ (henceforth, referred to as GSI) in the remainder of our experiments to minimize potential confounding effects. Based on the improved glucose tolerance phenotype of *L-Rbpj* mice, which lack hepatocyte Notch activity [14], we hypothesized that GSIs increased hepatic insulin sensitivity. Indeed, GSIs increased insulin-mediated phosphorylation of Akt and downstream targets (i.e., GSK3) in primary hepatocytes (not shown) and liver (Figure 1C and Supplemental Figure 1E). To determine if this effect was drug-specific or applicable across the class, we treated primary hepatocytes with a different but structurally similar γ-secretase inhibitor, Compound E (Supplemental Figure. 2), which we have previously shown to effectively block NICD generation in primary hepatocytes [14]. Consistent with *in vivo* effects of DBZ, *in vitro* application of Compound E reduced hepatocyte *Glucose-6-phosphatase* (*G6pc*) gene expression (Figure 1D), as well as glucose production in response to sub-pharmacologic insulin concentrations (Figure 1E). We next performed tracer-based glucose turnover studies in HFD-fed, GSI- or vehicle-treated mice. Despite unchanged body weight (not shown), GSI-treated mice showed reduced basal glucose (Figure 1F) and lower hepatic glucose production (Figure 1G), confirming the effect of GSIs to increase hepatic insulin sensitivity.

### 3.2 GSIs induce adipocyte insulin resistance, and reduce adipose glucose uptake

**Figure 2:**
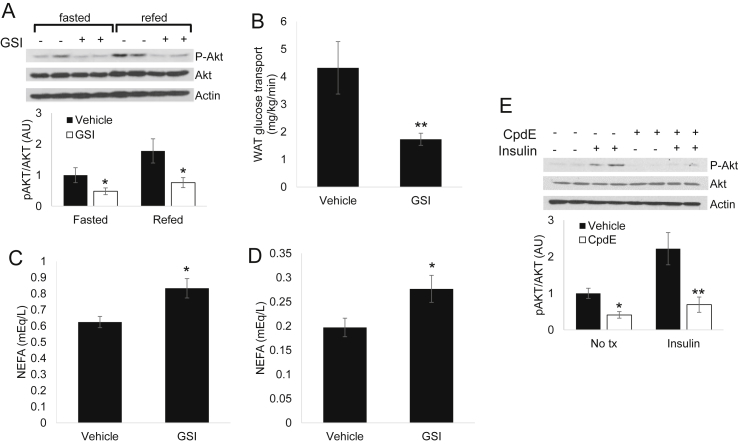
**GSIs decrease adipose insulin signaling and glucose uptake (A)** Western blots (top) and quantification of pAKT signal (normalized to total Akt signal, bottom) from eWAT of GSI-treated mice sacrificed after a 16 h fast or after a 16 h fast followed by 4 h refeeding. **(B)** Glucose uptake in epididymal white adipose tissue (eWAT) from HFD-fed C57/Bl6 mice after 5 daily doses of vehicle or GSI, prior to glucose turnover studies (n = 9–11 mice/group). **(C)** Plasma non-esterified fatty acid (NEFA) in vehicle or GSI-treated, HFD-fed C57/Bl6 mice sacrificed after an 5 h fast or **(D)** after a 16 h fast followed by 4 h refeeding. **(E)** Western blots (top) and quantification of pAKT signal (normalized to total signal, bottom) from 3T3-L1 adipocytes, with or without CpdE pre-treatment for 16 h then exposed to 100 nM insulin for 15 min prior to lysis. Data shown are representative of 3 independent experiments. *P < 0.05, **P < 0.01 and ***P < 0.001 vs. Vehicle.

These data suggest that GSI-mediated improvement in glucose tolerance is at least partially attributable to reduced hepatic glucose production but does not eliminate the possibility of extra-hepatic GSI effects. To test this, we evaluated insulin signaling in other insulin-sensitive tissues. Interestingly, while we observed no effect on insulin signaling in skeletal muscle, GSI treatment reduced fasting or refed Akt phosphorylation in epididymal (eWAT) and inguinal white adipose tissue (iWAT) depots (Figure 2A and not shown). To determine the physiologic consequence of this apparent reduction in insulin sensitivity, we examined glucose uptake in GSI-treated, HFD-fed wildtype mice. Consistent with the biochemical changes observed above, we found unchanged 2DOG uptake in gastrocnemius (not shown) but reduced eWAT glucose uptake (Figure 2B). Similarly, we observed higher NEFA levels in fasted and refed GSI-treated mice (Figure 2C, D), consistent with a specific reduction in adipose insulin signaling, which we also observed in cultured 3T3-L1 adipocytes (Figure 2E). Taken together, these data suggest that GSI-mediated improvement in whole-body glucose homeostasis is due to improved hepatic insulin sensitivity but mitigated in part by reduced adipose insulin signaling.

### 3.3 Adipose Notch signaling reflects both adipocyte and stromovascular contributions

**Figure 3:**
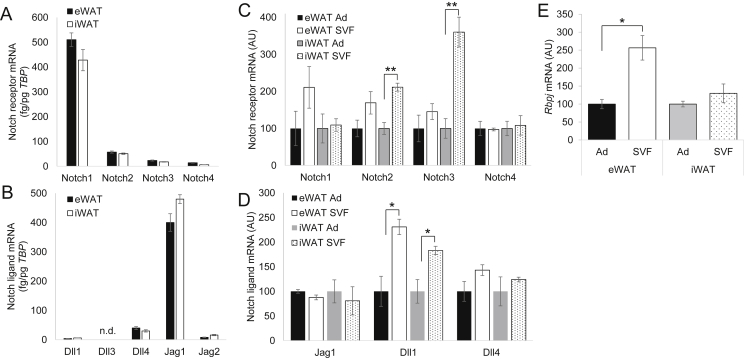
**Adipose Notch signaling is determined by adipocytes and stromovascular cells (A)** Notch receptor and **(B)** Notch ligand expression in epididymal white adipose tissue (eWAT) or inguinal white adipose tissue (iWAT) of chow-fed C57/Bl6 mice sacrificed after a 16 h fast. **(C)** Notch receptor, **(D)** ligand and **(E)** transcriptional effector (*Rbpj*) expression in floated adipocytes (Ad) and pelleted stromovascular fraction (SVF) isolated from eWAT and iWAT of chow-fed C57/Bl6 mice sacrificed after a 16 h fast. *P < 0.05 and **P < 0.01 as compared to the indicated control.

Although Notch has been shown to affect adipocyte differentiation [24], its potential role in adipose tissue homeostasis has only recently been postulated. To begin to study the potential role of Notch signaling in developed adipose, we surveyed Notch pathway expression in representative visceral (eWAT) and subcutaneous (iWAT) adipose tissue depots of adult mice. Of the four Notch receptors and five Notch ligands found in mammalian cells, *Notch1* and *Dll4*/*Jag1* represented the predominantly expressed adipose receptor and ligands, respectively (Figure 3A, B). Next, to determine sub-adipose expression patterns, we isolated primary adipocytes from the stromal vascular fraction (SVF) by collagenase treatment and centrifugation (Supplementary Figure. 3) and found that adipose Notch signaling arises from both adipocytes and SVF cells (Figure 3C, D), with relatively higher SVF contributions in visceral adipose depots.

### 3.4 Adipocyte-specific deletion of Rbp-Jk does not affect glucose homeostasis

**Figure 4:**
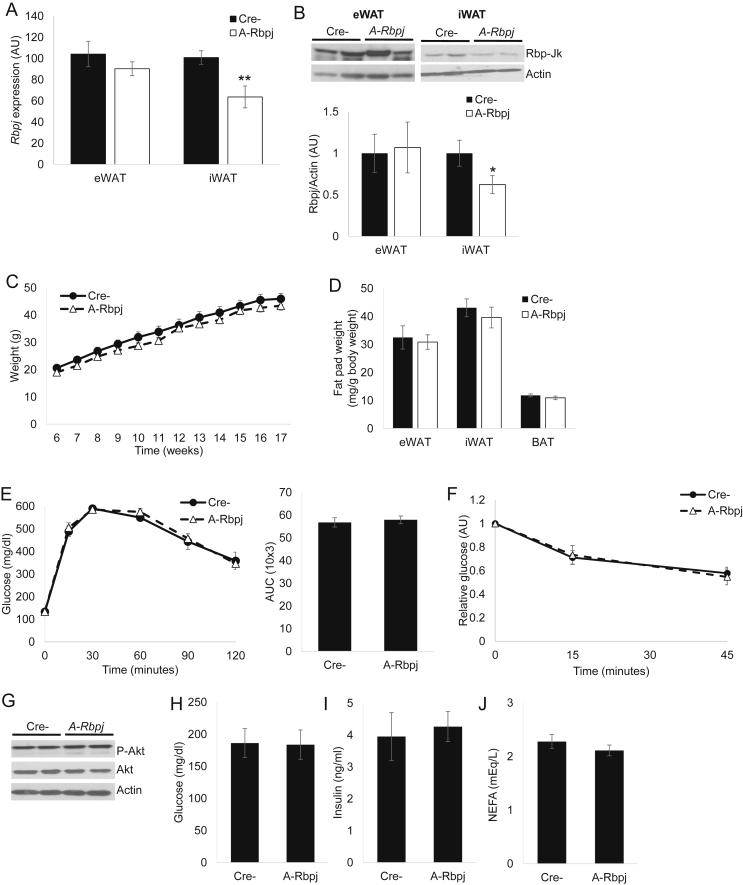
**Inhibition of adipocyte Notch signaling does not affect glucose homeostasis (A)***Rbpj* mRNA and **(B)** protein levels by Western blot (top) with quantification (normalized to Actin signal, bottom) in eWAT and iWAT of HFD-fed *A-Rbpj* and Cre- control mice sacrificed after a 16 h fast (n = 7 mice/group). **(C)** Body weight curve, **(D)** adipose depot weights, **(E)** GTT (left) and AUC during GTT (right), and **(F)** insulin tolerance testing (ITT) in HFD-fed *A-Rbpj* and control mice (n = 7 mice/group). **(G)** Western blots of eWAT isolated from HFD-fed *A-Rbpj* and control mice sacrificed after a 16 h fast, followed by 4 h refeeding. **(H)** Blood glucose, **(I)** plasma insulin and **(J)** NEFA levels in HFD-fed *A-Rbpj* and control mice sacrificed after a 16 h fast (n = 7 mice/group). *P < 0.05 vs. Cre- mice.

GSI-induced adipose insulin resistance could reflect cell-autonomous (adipocyte) Notch-dependent or –independent effects or a compensatory response to increased hepatic insulin sensitivity. We discarded the latter hypothesis due to decreased insulin sensitivity in GSI-treated 3T3-L1 adipocytes, but, to distinguish between the former, we generated adipocyte-specific Rbp-Jk (*A-Rbpj*) mice. Rbp-Jk is the common transcriptional effector of all 4 Notch receptors [25] and is expressed in both adipocytes and SVF cells (Figure 3E); genetic ablation using Adiponectin-Cre transgenic mice should result in a complete and specific loss of Notch activity in post-developmental adipocytes but leave intact the substantial SVF *Rbpj* expression. *A-Rbpj* mice were born at expected frequency, without obvious developmental abnormality, and consistent with Ad/SVF *Rbpj* expression patterns, had reduced iWAT but virtually unchanged *Rbpj* mRNA and protein levels in eWAT (Figure 4A, B). *A-Rbpj* mice showed similar weight gain on chow and HFD (not shown and Figure 4C) with similar adipose depot tissue weights as littermate controls (Figure 4D) as well as unchanged glucose tolerance (Figure 4E) and insulin sensitivity (Figure 4F). Consistently, *A-Rbpj* mice showed normal refeeding-induced Akt phosphorylation, fasted glucose/insulin and NEFA levels (Figure 4G–J). These data suggest that a specific reduction of Notch activity in developed adipocytes does not affect local or systemic insulin sensitivity.

### 3.5 Adipocyte-specific reduction of γ-secretase activity reduces adipose insulin sensitivity

**Figure 5:**
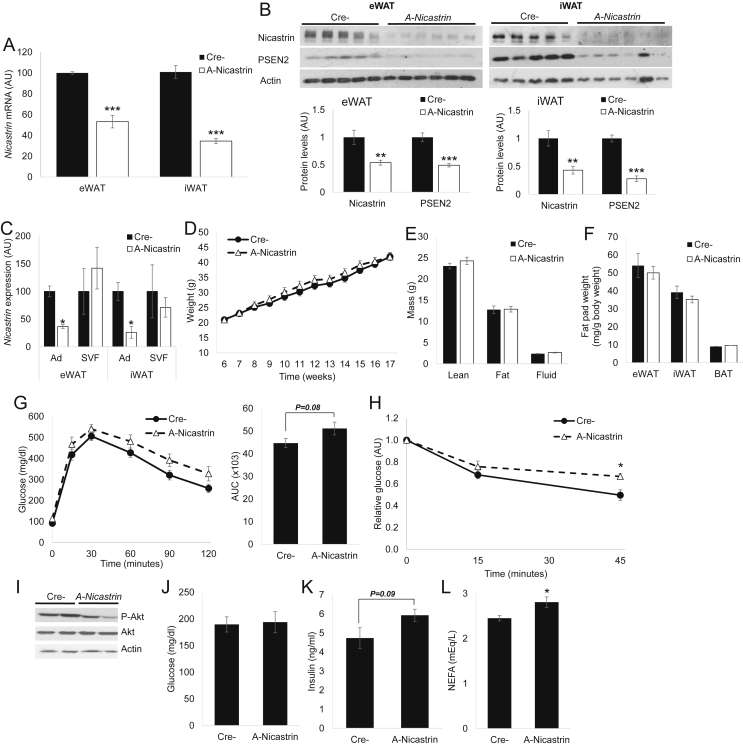
**Disruption of adipocyte γ-secretase reduces adipose insulin sensitivity (A)***Nicastrin* mRNA and **(B)** protein levels by Western blot (top) with quantification (normalized to Actin signal, bottom) in eWAT and iWAT of HFD-fed *A-Nicastrin* and Cre- control mice sacrificed after a 16 h fast (n = 7 mice/group). **(C)***Nicastrin* mRNA in floated adipocytes (Ad) and SVF isolated from eWAT and iWAT of HFD-fed *A-Nicastrin* and control mice sacrificed after a 16 h fast (n = 3 mice/group). **(D)** Body weight curve, **(E)** body composition, **(F)** adipose depot weights, **(G)** GTT (left) and AUC during GTT (right), and **(H)** ITT in HFD-fed *A-Nicastrin* and control mice (n = 7 mice/group). **(I)** Western blots of eWAT isolated from HFD-fed *A-Nicastrin* and control mice sacrificed after a 16 h fast, followed by 4 h refeeding. **(J)** Blood glucose, **(K)** plasma insulin and **(L)** NEFA levels in HFD-fed *A-Nicastrin* and control mice sacrificed after a 16 h fast (n = 7 mice/group). *P < 0.05 vs. Cre-mice.

We next hypothesized that GSI-induced reduction in adipose glucose disposal is due to Notch-independent means, but to prove cell-autonomous effects, we required a model of adipocyte-specific γ-secretase deficiency. Nicastrin is the obligate targeting component of the γ-secretase enzyme complex [26], and unlike other components (Presenilin 1/2, Aph1a/b), is non-redundant [10]. Nicastrin expression is ubiquitous [27] and equally abundant in adipocytes and stromovascular cells (not shown). As such, we generated adipocyte-specific Nicastrin (*A-Nicastrin*) knockout mice, which demonstrated lower *Nicastrin* mRNA and protein levels in both eWAT and iWAT (Figure 5A, B) with specific reductions in the adipocyte fractions of adipose tissue from these mice (Figure 5C). Expectedly, given the necessity of Nicastrin for γ-secretase stability and activity [28], [29], C-terminal fragment levels of Presenilin 1 and 2 were lower in *A-Nicastrin* mice (not shown and Figure 5B). *A-Nicastrin* mice were born at Mendelian frequency, without gross developmental phenotype and unchanged body weight and adiposity with chow- (Supplemental Figure 4A, B) or HFD-feeding (Figure 5D–F), but in contrast to *A-Rbpj* mice, *A-Nicastrin* mice showed a trend towards reduced glucose intolerance and insulin sensitivity as compared to Cre- controls (Supplemental Figure 4C, G, H). Consistently, Akt phosphorylation was reduced in eWAT and iWAT from HFD-fed *A-Nicastrin* mice (Figure 5I and not shown), which also showed a relative hyperinsulinemia and excess circulating fatty acids (Figure 5J–L) resulting in a trend towards increased liver weight and triglyceride content (Supplementary Figure. 5). In sum, these data prove that the γ-secretase complex, but not canonical Notch signaling, regulates adipocyte insulin sensitivity to impact systemic glucose homeostasis.

## 4 Discussion

Several *in vitro* studies in 3T3-L1 adipocytes and adipose-derived stem cells have shown that either constitutive activation or reduction of Notch activity can inhibit normal adipogenesis [24], [30]. This is not as paradoxical as it seems and is in fact consistent with normal Notch control of lateral inhibition and the proliferation/differentiation decision tree [3]. Probably the best-characterized example of this is the necessity for sequential Notch activation, then inactivation, in endocrine lineage specification prior to pancreatic β-cell development [31]. Further complexity is introduced by the interplay of downstream targets of Notch signaling, as Hes/Hey proteins are transcriptional regulators in their own right, with potential modulatory effects on differentiation [32].

These intricate layers of Notch regulation are required to ensure proper cell-fate decision and normal tissue architecture [33] but present challenges when designing mouse experiments to understand the function of Notch signaling in the post-development state. Our approach circumvents this problem as Adiponectin-Cre acts only in mature adipocytes [18] and allows for mostly intact *de novo* differentiation of pre-adipocytes [34]. This is in contrast with models that utilize aP2-Cre, which has potential off-target (macrophage) as well as on-target but “early” effects in immature adipocytes [18], [35], [36], [37]. These differences likely explain the null phenotype of *A-Rbpj* mice observed in our study, as compared to the recently reported decreased body weight/adiposity and resultant improvement in glucose tolerance in *aP2-cre/Notch1*^flox/flox^ and *aP2-cre/Rbpj*^flox/flox^ animals [17]. We hypothesize aP2-Cre-mediated Notch loss-of-function results in impaired adipogenesis and/or macrophage function, consistent with effects of anti-DLL4 monoclonal antibody treatment, which inhibits macrophage Notch signaling, leading to decreased adiposity and consequent improvements in systemic insulin sensitivity [16]. Similarly, our work is in seeming contradiction with glucose intolerance in mice with constitutive adipocyte Notch activity (*Adiponectin-cre/NICD*^flox/flox^) [17], [38]. This mouse model should be interpreted with some caution, however, as constitutive Notch activation is unlikely to reflect pathophysiology. In fact, as predicted by altered adipogenesis seen in earlier 3T3-L1 studies, constitutive Notch activation results in lipodystrophy – markedly reduced adiposity but increased body weight due to hepatomegaly [38] – which is the likely proximal cause of the glucose intolerance and other metabolic disturbances seen with this model. The mechanism underlying the loss of adiposity with this Notch gain-of-function is likely multifactorial as these mice showed repression of adipogenic and lipogenic pathways with parallel reductions in subcutaneous adipose “browning”. Interestingly, this reduction in fat mass recapitulates the phenotype observed in aP2-driven Notch loss-of-function, albeit by different proposed mechanisms [17], [38], underscoring the difficulties in dissecting the perhaps parallel requirements for Notch signaling in developmental and homeostatic processes. There is opportunity, however, in the divergences observed in our study as compared to the published literature. For example, understanding whether temporal vs. cellular Cre “leakiness” explains metabolic benefit in aP2-driven Notch deficiency models, may uncover novel biology with therapeutic possibility, as evidenced by the proposed role of Notch signaling in browning of subcutaneous adipose depots [17]. By contrast, we do not observe altered gene expression of browning markers or substantive changes by staining in *Adiponectin-cre/Rbpj*^flox/flox^ or *Adiponectin-cre/Nicastrin*^flox/flox^ mice (data not shown).

In sum, our data demonstrate that Notch likely does not play an active role in maintenance of adipocyte function or local/systemic insulin sensitivity, although we cannot completely exclude potential Rbp-Jk-independent Notch activity [39], or that Adiponectin-cre-mediated recombination was not fully efficient. On the contrary, γ-secretase does appear to sensitize adipocytes to insulin action, by both biochemical (insulin signaling) and pharmacologic (insulin tolerance testing) proofs. That these effects do not markedly affect glucose tolerance is not unexpected given the relatively minor contribution of adipose to overall glucose disposal. This by no means argues against the importance of adipose in regulation of systemic insulin sensitivity, convincingly shown using genetic mouse models of adipocyte-specific gain- or loss-of-function of insulin signaling genes, such as the adipose-specific insulin receptor knockout [40]. Similarly, selective enhancement of adipocyte insulin sensitivity, by prolonging insulin action through knockout of the PTEN phosphatase, is sufficient to improve systemic glucose tolerance [41], whereas increased E4orf1 expression leads to impaired adipocyte insulin sensitivity and commensurate systemic effects [42], with reciprocal changes in plasma levels of the adipokine adiponectin (notably unaffected in *A-Nicastrin* mice, not shown) known to increase hepatic insulin sensitivity [43]. Finally, adipocytes additionally exert various indirect effects on whole-body glucose homeostasis by increasing lipid flux to various tissues, notably liver [44], [45]. In fact, the trend towards hepatic lipid content in *A-Nicastrin* mice may result from increased fatty acid flux to the liver that may be masked by increased insulin sensitivity in GSI-treated mice.

Although the specific γ-secretase target underlying altered adipose insulin sensitivity in GSI-treated or *A-Nicastrin* mice requires further study, our findings suggest that the beneficial effects observed in mice treated with GSIs or other Notch inhibitors [14], [15], [16] are likely mediated through effects on liver. Further, these and other data [46], [47], [48] suggest that Notch has distinct, tissue-specific roles in the post-developmental state, which likely relate to the capacity or necessity for cellular proliferation and differentiation in response to obesity or other injurious stimuli. Finally, our data predict that specific Notch inhibitors, such as monoclonal antibodies to receptors/ligands [16], [49], [50] or “decoy” receptors [15], [51] in clinical development for cancer, perhaps selected by dint of preferential hepatic Notch receptor/ligand antagonism [52], are likely to fare better for metabolic repurposing than non-specific inhibitors.

## Author contributions

D.P.S., A.B. and U.B.P designed the experiments, analyzed the data and wrote the manuscript. D.P.S., J.Y., K.K., C.Z. and S.B. performed the experiments.

## Funding

This work was funded in part by National Institutes of Health grants DK093604 (U.B.P.) and T32DK065522 (P.I.: S.E. Oberfield, support to D.P.S.), as well as Marilyn Fishman Grant for Diabetes Research from the Endocrine Fellows Foundation and a Research Fellowship award from the Pediatric Endocrine Society (D.P.S.) and a grant from the German Research Foundation BI1292/4-2 (A.L.B.). Funding sources had no involvement in collection, analysis and interpretation of data, in the writing of the report, or in the decision to submit the article for publication.

## Appendix A Supplementary data

**Supplementary Figure 1: DBZ induces dose-dependent intestinal metaplasia (a)** Periodic anti-Schiff (PAS) staining of small intestine and **(b)** quantification of PAS (+) goblet cells/villus in HFD-fed, C57/BL6 mice treated with 5 daily doses of Vehicle, DBZ 2mpk or DBZ 10mpk, sacrificed after a 16 h fast. **(c)** Body weight change from baseline and **(d)** epididymal WAT (eWAT) weight, normalized to total body weight in vehicle, DBZ 2mpk or DBZ 10mpk-treated mice (n=6 mice/group). **(e)** Western blots from livers of Vehicle- or GSI (DBZ 2mpk)-treated, C57/BL6 mice sacrificed after acute (5 minute) insulin exposure following a 16 h fast. ****P* < 0.001 vs. Vehicle.

**Supplementary Figure 2: Chemical structures of GSIs** Compound E and dibenzazepine (DBZ) were used for *in vitro* and *in vivo* experiments, respectively.

**Supplementary Figure 3: Adipocyte/SVF isolation** Adipocyte (*Adiponectin*) and macrophage (*Emr1*) marker expression in floated adipocytes (Ad) and pelleted stromovascular fraction (SVF) isolated from eWAT and iWAT of chow-fed C57/Bl6 mice sacrificed after a 16 h fast. **P* < 0.05 ***P* < 0.01, and ****P* < 0.001 as compared to the indicated control.

**Supplementary Figure 4: Disruption of adipocyte γ-secretase does not affect body weight or composition (a)** Body weight curve, **(b)** body composition and **(c)** GTT (left) and AUC during GTT (right) in chow-fed *A-Nicastrin* and Cre- control mice (n=6 mice/group).

**Supplementary Figure 5: Disruption of adipocyte γ-secretase does not significantly affect hepatic lipid content (a)** Liver weight, **(b)** lipid content and **(c)** histology in HFD-fed *A-Nicastrin* and Cre- control mice sacrificed after a 16 h fast (n=7 mice/group).
