# Supplementary material for: Adipocyte-specific blockade of gamma-secretase, but not inhibition of Notch activity, reduces adipose insulin sensitivity
Source: Mol Metab. 2015 Dec 2;5(2):113–21. doi: 10.1016/j.molmet.2015.11.006 (PMC4735659; doi:10.1016/j.molmet.2015.11.006)

Supplementary Figure 1

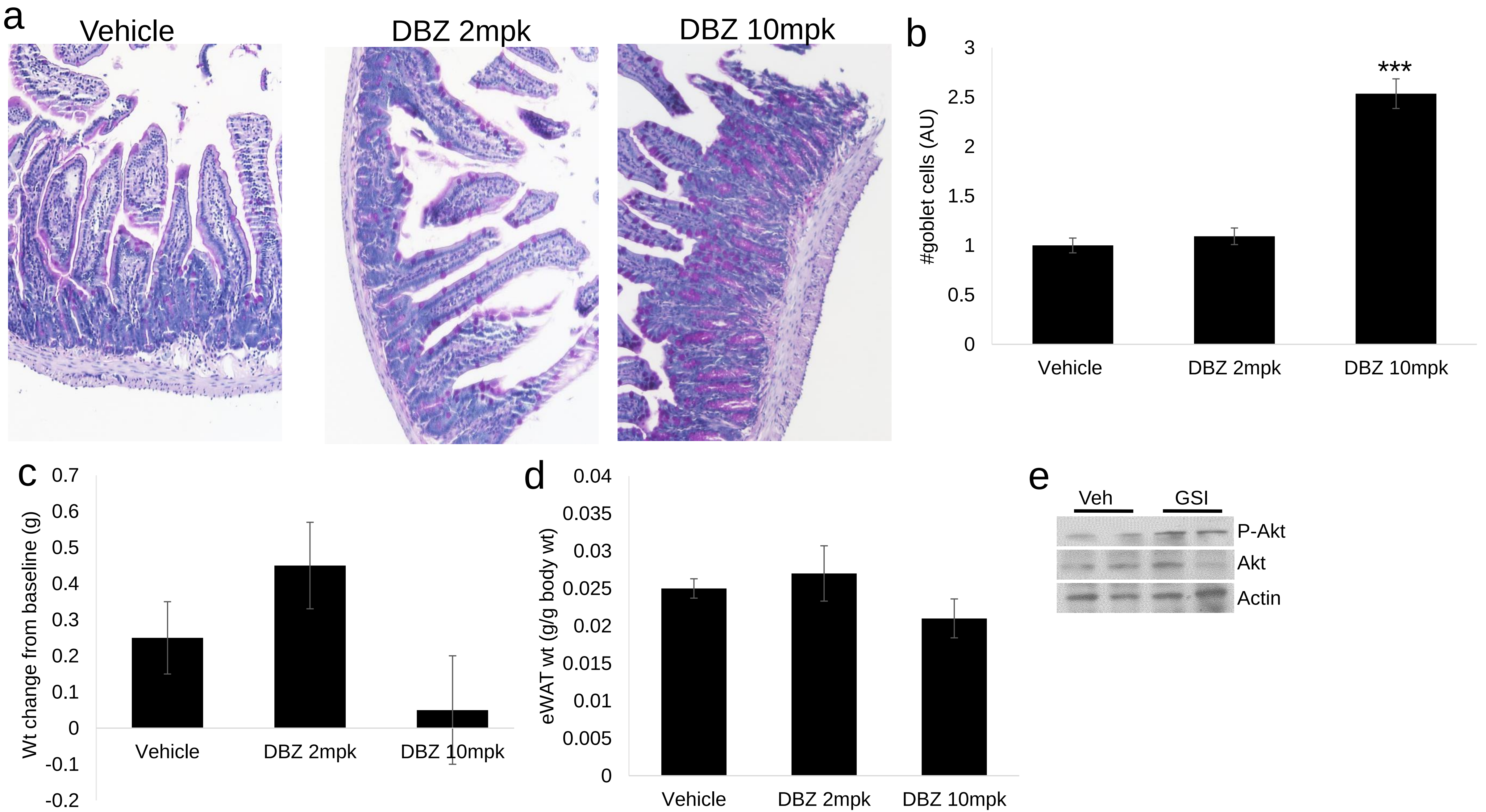

Supplementary Figure 2

Compound E

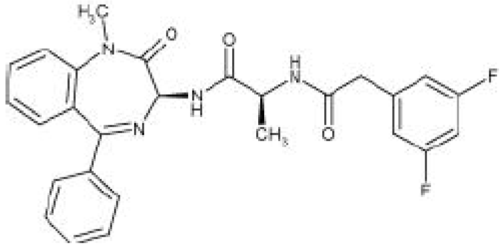

DBZ

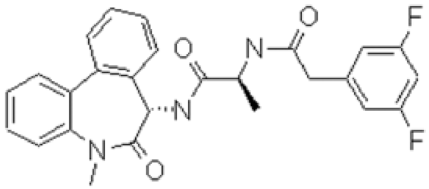

Supplementary Figure 3

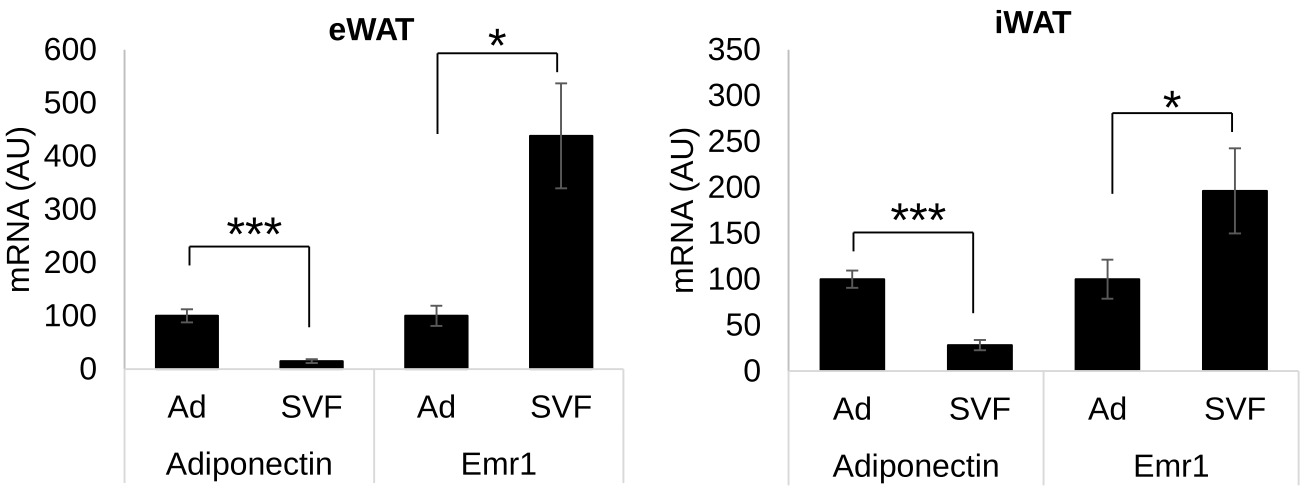

Supplementary Figure 4

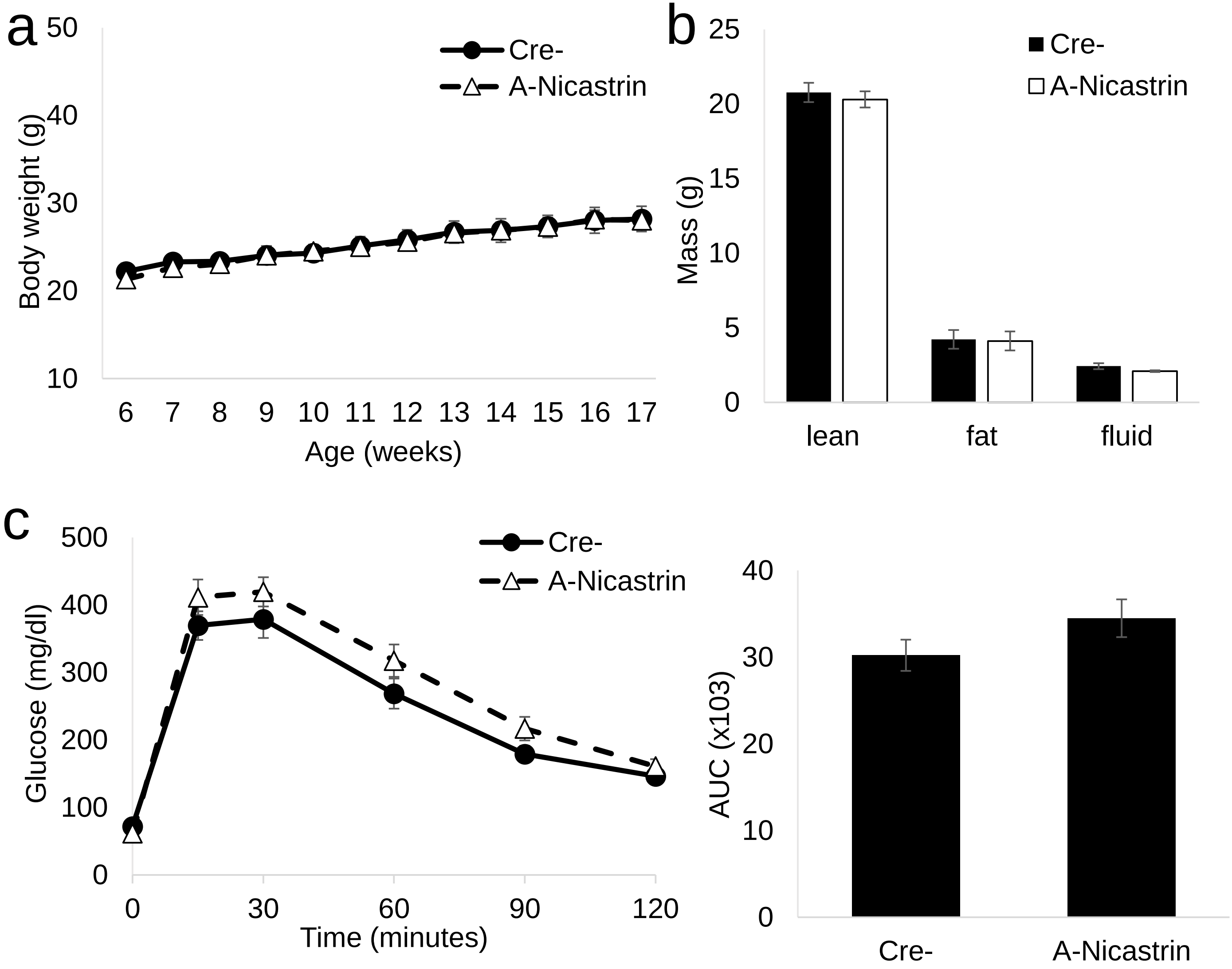

Supplementary Figure 5

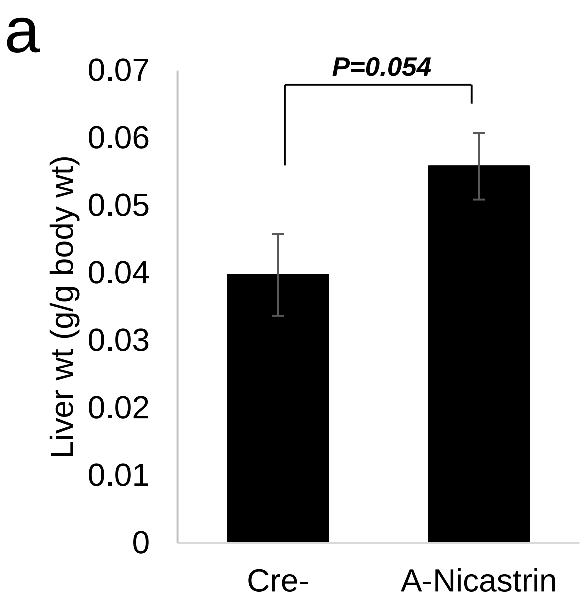

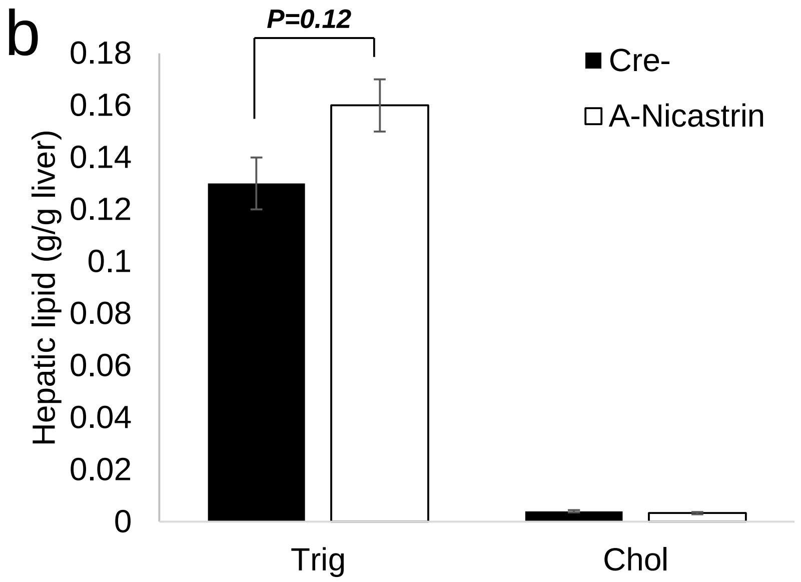

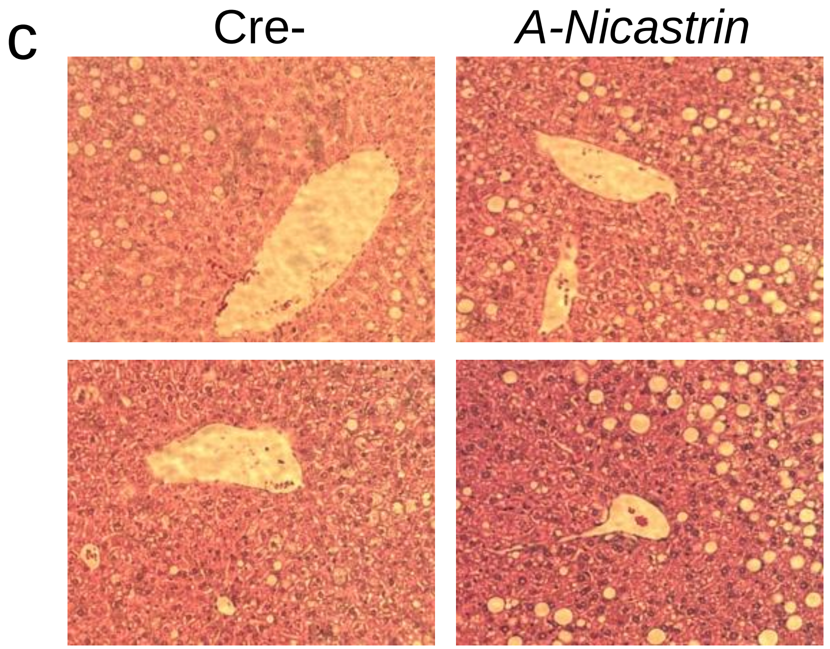

Supplement: Supplementary file 1 [file mmc1.pdf]
